# The Protective Effects of Alpha-Lipoic Acid and Coenzyme Q_10_ Combination on Ovarian Ischemia-Reperfusion Injury: An Experimental Study

**DOI:** 10.1155/2016/3415046

**Published:** 2016-08-15

**Authors:** Ahmet Ali Tuncer, Mehmet Fatih Bozkurt, Tulay Koken, Nurhan Dogan, Mine Kanat Pektaş, Didem Baskin Embleton

**Affiliations:** ^1^Department of Pediatric Surgery, Afyon Kocatepe University Hospital, 03000 Afyonkarahisar, Turkey; ^2^Department of Pathology, Faculty of Veterinary Medicine, Afyon Kocatepe University, 03000 Afyonkarahisar, Turkey; ^3^Department of Biochemistry, Afyon Kocatepe University Medical Faculty, 03000 Afyonkarahisar, Turkey; ^4^Department of Biostatistics, Afyon Kocatepe University Medical Faculty, 03000 Afyonkarahisar, Turkey; ^5^Department of Obstetrics & Gynecology, Afyon Kocatepe University Hospital, 03000 Afyonkarahisar, Turkey

## Abstract

*Objective*. This study aims to evaluate whether alpha-lipoic acid and/or coenzyme Q_10_ can protect the prepubertal ovarian tissue from ischemia-reperfusion injury in an experimental rat model of ovarian torsion.* Materials and Methods*. Forty-two female preadolescent Wistar-Albino rats were divided into 6 equal groups randomly. The sham group had laparotomy without torsion; the other groups had torsion/detorsion procedure. After undergoing torsion, group 2 received saline, group 3 received olive oil, group 4 received alpha-lipoic acid, group 5 received coenzyme Q_10_, and group 6 received both alpha-lipoic acid and coenzyme Q_10_ orally. The oxidant-antioxidant statuses of these groups were compared using biochemical measurement of oxidized/reduced glutathione, glutathione peroxidase and malondialdehyde, pathological evaluation of damage and apoptosis within the ovarian tissue, and immunohistochemical assessment of nitric oxide synthase.* Results*. The left ovaries of the alpha-lipoic acid + coenzyme Q_10_ group had significantly lower apoptosis scores and significantly higher nitric oxide synthase content than the left ovaries of the control groups. The alpha-lipoic acid + coenzyme Q_10_ group had significantly higher glutathione peroxidase levels and serum malondialdehyde concentrations than the sham group.* Conclusions*. The combination of alpha-lipoic acid and coenzyme Q_10_ has beneficial effects on oxidative stress induced by ischemia-reperfusion injury related to ovarian torsion.

## 1. Introduction

Ovarian torsion is defined as the total or partial rotation of the ovary, the fallopian tube, or both, around its vascular axis. This clinical situation is an emergency of pediatric surgery and gynecology as it causes abdominal pain which requires surgery. Therefore, detorsion of the ovary is surgically performed to maintain its proper circulation [1, 2].

Ovarian torsion and detorsion (T/D) induce ischemia-reperfusion injury which leads to the occurrence of morphological, histological, and biochemical alterations within the ovarian tissue. This is due to a pathological process, which is named ischemia-reperfusion injury. As the ischemic tissue receives an excessive supply of molecular oxygen during reperfusion, acute ischemic injury is worsened by the products of oxidative stress including free radicals and oxygen reactive species. These products cause lipid peroxidation which impairs the permeability of cell membranes and disrupts the integrity of cells [3–6].

Alpha-lipoic acid (ALA) is found naturally within the mitochondria. It is an essential cofactor for pyruvate dehydrogenase and alpha-ketoglutarate dehydrogenase. The coupling of ALA and its reduced form (dihydrolipoic acid) is described as the “ideal,” “unique,” and “universal antioxidant” [7]. It has been demonstrated that ALA protects various membrane systems from oxidative injury including neuronal membranes, erythrocyte membranes, and mitochondrial membranes [8–10]. The efficiency of ALA has been shown in diabetes mellitus, atherosclerosis, ischemia-reperfusion diseases, multiple sclerosis, cognitive losses, and senile dementia [11].

Coenzyme Q_10_ (CoQ_10_) is a strong antioxidant which provides stabilization of cell membranes by participating in the mitochondrial electron transport chain. This molecule also acts as a cofactor in the synthesis of adenosine triphosphate (ATP) by oxidative phosphorylation. It has been shown that CoQ_10_ inhibits lipid peroxidation, scavenges free oxygen radicals, and increases the utilization of oxygen in energy production [12–14].

Free radicals which occur as a result of ischemia-reperfusion and oxidative stress affect the phospholipids on the cell membrane and they create phosphoryl choline and ceramide on the scale of neutral sphingomyelinase (n-SMase). Ceramide leads to caspase activation and activates apoptosis. CoQ_10_ decreases apoptosis by affecting both the creation of free radicals and n-SMase step. Likewise, alpha-lipoic acid prevents apoptosis both by sweeping free oxygen radicals as an antioxidant and by affecting the caspase system [15, 16].

The present study aims to evaluate whether ALA, CoQ_10_, or the combination of both can be used to protect prepubertal ovarian tissue from ischemia-reperfusion injury in an experimental rat model of ovarian torsion.

## 2. Materials and Methods

This study was approved by the Ethical Committee of Afyon Kocatepe University for Animal Experiments (number: 59269667/281, date: 15.02.2013). The experiments in this study were performed in accordance with the guidelines for animal research.

### 2.1. Study Design

The rats that were included in the experiment had intact circadian rhythm and ad libitum feeding within their natural environment. Forty-two female preadolescent Wistar-Albino rats (mean age: 4 weeks, weight range: 40–45 g) were divided into 6 equal groups randomly.

All surgical operations were performed under intramuscular 50 mg/kg ketamine (Ketasol 10%, Richter Pharma AG, Wels, Austria) and 10 mg/kg xylazine (Alfazyne 2%, Alfasan International BV, Woerden, Netherlands) anesthesia. Rats were placed in the dorsal recumbent position. A 1.5 cm midline laparotomy incision was made under sterile conditions so that the abdomen was opened and the large intestines were separated gently. In order to visualize the left ovary, large intestines are placed on the left side of the rat's abdomen. After the left ovary was found, it was made to undergo torsion of 720 degrees clockwise and then fixated to the anterior abdominal wall with a single 5/0 prolene suture. Afterwards, the abdominal incision was repaired with continuous sutures. Rats were covered with gauze to be protected from hypothermia and they recovered from the effects of anesthesia in approximately 30 minutes. Medication was given orally with gavage after an hour postoperatively; detorsion was performed at the 3rd hour and bilateral oophorectomy and blood samples were taken at the 9th hour postoperatively. The reason for choosing postoperative 9th hour for biochemical, histopathological, and immunohistochemical evaluation is based on pharmacokinetic properties of the molecules. When ALA is taken orally, it is rapidly absorbed and reaches plasma peak levels in 30 minutes to 1 hour [17]. In contrast, coenzyme Q_10_ reaches peak plasma levels in about 4–6 hours after its oral administration [18].

Group 1 (sham group) included 7 rats that had laparotomy without torsion. Group 2 (saline group) consisted of 7 rats that underwent T/D and received saline orally (Polifleks 0.9% NaCl, Eczacıbaşı-Baxter, Istanbul, Turkey). Group 3 (olive oil group) included 7 rats that had T/D and received olive oil orally (Taris, Izmir, Turkey). Group 4 (ALA group) consisted of 7 rats that underwent T/D and received ALA orally (100 *μ*M/kg/day, ≥99% titration in olive oil, Sigma-Aldrich Chemie GmbH, Steinheim, Germany). Group 5 (CoQ_10_ group) included 7 rats that had T/D and received CoQ_10_ orally (10 mg/kg/day, ≥98% titration in olive oil, Sigma-Aldrich Chemie GmbH, Steinheim, Germany). Group 6 (ALA + CoQ_10_) included 7 rats that underwent T/D and received both ALA and CoQ_10_ orally. As both ALA and CoQ_10_ were administered in olive oil solutions in the treatment groups, an olive oil group was included to rule out whether olive oil itself would exert antioxidant effects.

### 2.2. Biochemical Analysis for Oxidant-Antioxidant Status

Blood samples were collected into heparinized tubes and were examined in the biochemistry laboratory of the study center. Serum levels of oxidized/reduced glutathione (GSH) and malondialdehyde (MDA) concentrations were determined by high-performance liquid chromatograph (HPLC) in the isocratic phase in the Agilent 1100 series instrument with fluorescent detection (Ex:515, Em:553 nm for MDA; Ex:385, Em:515 for GSH) using a kit from Chromsystems Chemicals GmbH (glutathione kits, Chromsystems Chemicals, Munich, Germany; malondialdehyde kits, Chromsystems Chemicals, Munich, Germany). The results were evaluated as *µ*mol/g hemoglobin for GSH and *µ*mol/L hemoglobin for MDA.

Serum levels of glutathione peroxidase were determined using glutathione peroxidase kits (glutathione peroxidase assay kits, Cayman Chemicals, Ann Arbor, USA). ELISA microanalyzer was used for absorbance assays (ChemWell 2910, Awareness Technology Inc., Palm City, USA). The results were evaluated as U/g hemoglobin for glutathione peroxidase.

### 2.3. Histopathological Evaluation

Since the study was carried out on ovarian tissues of preadolescent rats, the pathologist was unable to evaluate the ovarian reserve based on the counts of primordial, preantral, and antral follicles. In order to specify the ovarian damage histopathologically, at least five microscopic sections were examined and semiquantitative scores were obtained. The presence of follicular cell degeneration, hyperemia, hemorrhage, and inflammation were used as the criteria for ovarian injury. Each specimen was scored using a scale ranging from 0 to 3 (0: none; 1: mild; 2: moderate; 3: severe) [19]. The ovarian sections were analyzed by the same pathologist who was blinded to experiment groups.

### 2.4. TUNEL Assay for Apoptosis

Paraffin blocks were prepared for histopathological examination and 5 *µ*m sections from these blocks were transferred to positive charged slides for TUNEL assay. Nuclease-free proteinase K (0.6 units/mL, pH 8.0) incubation was applied (in 37°C oven, for 10 minutes) for antigen retrieval. After washing with PBS, 10-minute 3% hydrogen peroxide application was made at room temperature for blocking of endogenous peroxidase activities. Sections were transferred into humidity chamber and a commercial apoptosis kit (In Situ Cell Death Detection Kit, POD (cat. number 1 684 817), Roche Diagnostics GmbH, Mannheim, Germany) was applied according to the manufacturer's procedure: slides are incubated in TUNEL mixture for 1 hour at 37°C and then they are incubated for 30 minutes in converter pod at 37°C. All slides were stained with AEC chromogen. For counterstain, Mayer's hematoxylin staining was performed. After each step, sections were washed with PBS. The immunoreactivity for apoptosis was graded from 0 to 4 with a light microscope in terms of density and distribution of staining: (0) no positivity on stroma and follicles, (1) mild positivity only in follicles, (2) moderate positivity in follicles and slight positivity in stroma, (3) moderate positivity in follicles and stroma, and (4) severe positivity in follicles and stroma.

### 2.5. Immunohistochemical Assay for Nitric Oxide Synthase (NOS)

Streptavidin-biotin-peroxidase complex (VECTASTAIN Elite ABC Kit PK-6101, Vector Laboratories, Peterborough, UK) method was used. Briefly, 5 *μ*m tissue sections were mounted on positive charged slides from paraffin blocks. Deparaffinized and rehydrated sections were transferred into 3% hydrogen peroxide for blocking endogenous peroxides and then antigen retrieving processes were carried out. Nonspecific immunoglobulins were blocked with nonimmune sera. Antibodies against NOS (C-terminal-polyclonal 1/40 dilution (ab15203), Abcam, Cambridge, UK) were applied to the sections. Then, biotinylated secondary antibody and streptavidin-peroxidase were dropped on the tissue sections. After this process, sections were visualized with 3-amino-9-ethylcarbazole chromogen (AEC substrate kit (code number 00-2007), Invitrogen, Basel, Switzerland). The immunoreactivity for NOS was graded from 0 to 4 with a light microscope in terms of density and distribution of staining: (0) no positivity in stroma and follicles, (1) mild positivity in follicles, (2) moderate positivity in follicles and slight positivity in stroma, (3) moderate positivity in follicles and stroma, and (4) severe positivity in follicles and stroma.

### 2.6. Statistical Analysis

Collected data were analyzed by MedCalc (version 12.7.5.0, Ghent, Belgium). Continuous variables were expressed as mean ± standard deviation (range: minimum–maximum) whereas categorical variables were denoted as mean rank. Kruskal-Wallis test and Mann-Whitney *U* test were used for the comparisons. Conover-Inman post hoc test was used to determine the groups between which statistical significance exists. Two-tailed *P* values less than 0.05 were accepted to be statistically significant.

## 3. Results

**Figure 1:**
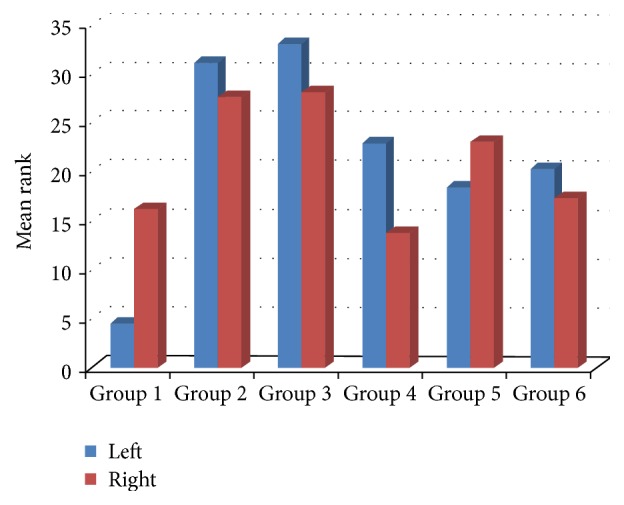
Mean rank of apoptosis.

**Figure 2:**
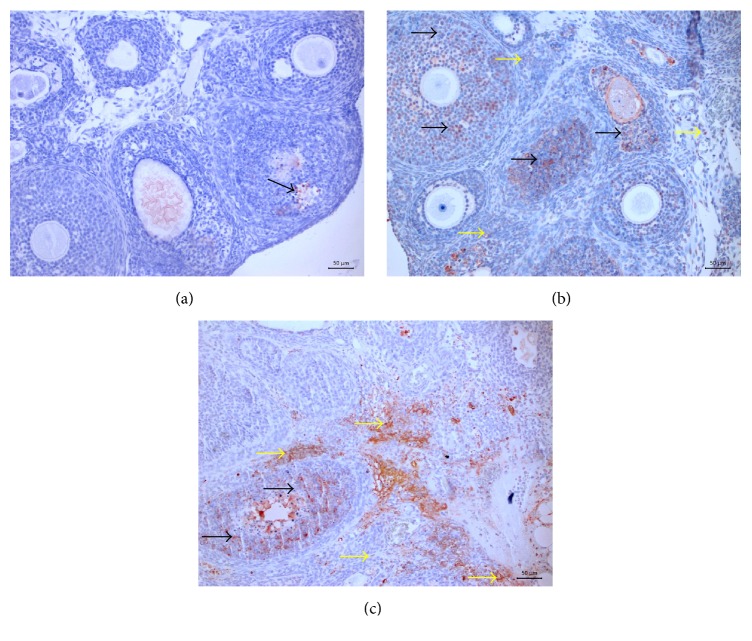
Slight TUNEL positivity in follicular cells (arrow) in group 1 (a). Moderate TUNEL positivity in follicular cells (arrows) and slight positivity in stroma (yellow arrows) in group 6 (b). Severe TUNEL positivity in follicular cells (arrows) and stroma (yellow arrows) in group 2 (c). ×50. TUNEL method. AEC chromogen. Gill's (I) Hematoxylin.

Table 1 compares the pathological scoring of the sham, control, and study groups (Table 1). When compared to the right ovaries, the left ovaries had significantly higher apoptosis scores in the saline, olive oil, ALA, CoQ_10_, and ALA + CoQ_10_ groups (*P* = 0.003, *P* = 0.002, *P* = 0.001, *P* = 0.002, and *P* = 0.008, resp.). The right ovaries of the sham, control, and study groups were statistically similar in aspect of apoptosis scores (*P* = 0.07). The left ovaries of the ALA + CoQ_10_ group had significantly lower apoptosis scores than the left ovaries of the saline and olive oil groups (*P* = 0.001 for both) (Figures 1 and 2). The ALA, CoQ_10_, and ALA + CoQ_10_ groups had statistically similar apoptosis scores (*P*_4-5_ = 0.430, *P*_4-6_ = 0.556, and *P*_5-6_ = 0.612).

**Figure 3:**
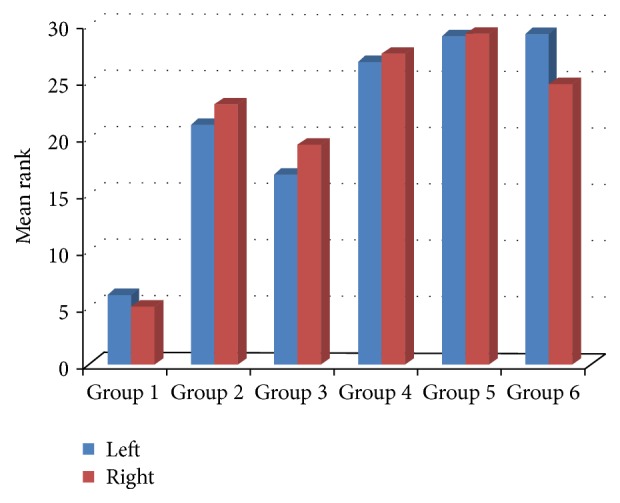
Mean rank of NOS immunoreactivity.

**Figure 4:**
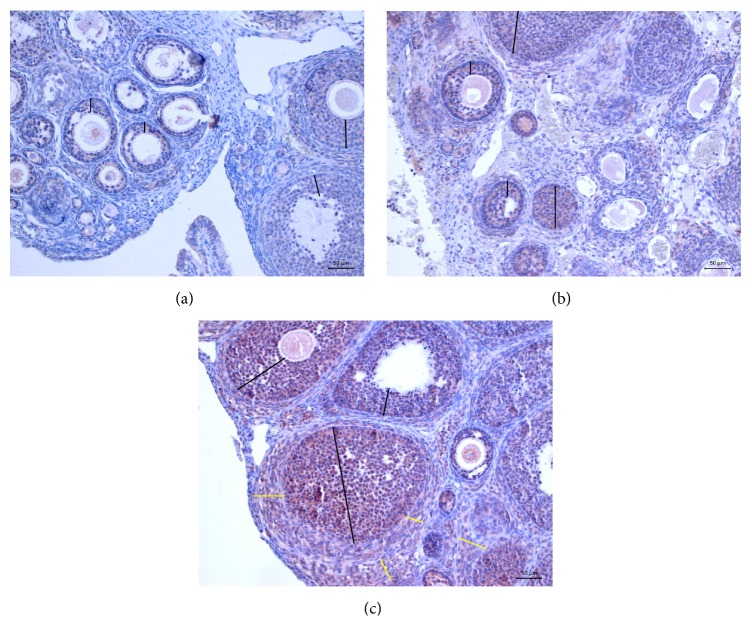
Slight NOS positivity in follicular cells (lines) (a). Moderate NOS positivity in follicular cells in group 3 (lines) (b). Severe NOS positivity in follicular cells (lines) and stroma (yellow lines) in group 6 (c). ×50. ABC peroxidase. AEC chromogen. Gill's (I) Hematoxylin.

The right and left ovaries had statistically similar NOS level in the sham, control, and study groups (*P* = 0.530, *P* = 0.644, *P* = 0.367, *P* = 0.709, *P* = 0.947, and *P* = 0.578, resp.). The right ovaries of the ALA + CoQ_10_ group had significantly higher NOS content than the right ovaries of the saline and olive oil groups (*P* = 0.001 for both). The left ovaries of the ALA + CoQ_10_ group had significantly higher NOS content than the left ovaries of the saline and olive oil groups (*P* = 0.001 for both) (Figures 3 and 4). The ALA, CoQ_10_, and ALA + CoQ_10_ groups had statistically similar NOS content but NOS content of the ALA + CoQ_10_ group tended to be higher than those of the ALA and CoQ_10_ groups (*P*_4-5_ = 0.126, *P*_4-6_ = 0.248, and *P*_5-6_ = 0.378).

**Figure 5:**
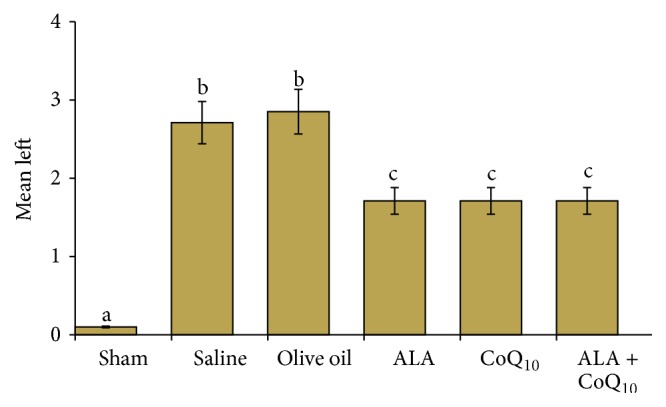
When compared with the left ovaries of the saline and olive oil groups, the left ovaries of the alpha-lipoic acid, coenzyme Q_10_, and alpha-lipoic acid + coenzyme Q_10_ groups had significantly lower ovarian damage scores (*P* = 0.03). The same letters indicated that the difference was statistically insignificant but different letters showed that there was statistical significance between the groups.

When compared with the left ovaries of the saline and olive oil groups, the left ovaries of the ALA, CoQ_10_, and ALA + CoQ_10_ groups had significantly lower ovarian damage scores (*P* = 0.03). However, there were no statistically significant differences among the study groups (*P*_4-5_ = 0.1, *P*_4-6_ = 1.00, and *P*_5-6_ = 0.1) (Figure 5). The right and left ovaries of the sham group were statistically similar whereas the left ovaries of the remaining groups had significantly higher tissue damage scores than those of the contralateral ovaries (*P* values are enlisted in Table 1).

**Figure 6:**
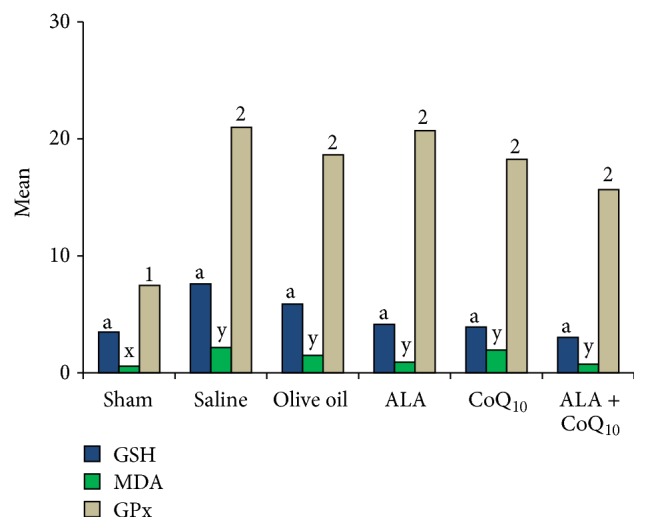
When compared with the sham group, the alpha-lipoic acid + coenzyme Q_10_ group had significantly higher glutathione peroxidase levels and serum malondialdehyde concentrations (*P* = 0.007 and *P* = 0.027, resp.). When compared with the control group, the alpha-lipoic acid + coenzyme Q_10_ group had lower serum malondialdehyde concentrations (*P* = 0.05). The same letters indicated that the difference was statistically insignificant but different letters showed that there was statistical significance between groups (“a” for glutathione; “1, 2” for malondialdehyde; and “x, y” for glutathione peroxidase).

The biochemical assessments of the oxidant-antioxidant statuses of the sham, control, and study groups were compared (Figure 6). All six groups were statistically similar with respect to serum glutathione concentrations (*P* = 0.069). When compared with the sham group, the ALA + CoQ_10_ group had significantly higher glutathione peroxidase levels and serum malondialdehyde concentrations (*P* = 0.007 and *P* = 0.027, resp.). There were no statistically significant differences between the glutathione peroxidase levels of the control groups (groups 2 and 3) and the ALA, CoQ_10_, and ALA + CoQ_10_ groups (*P*_2-4_ = 0.687, *P*_2-5_ = 0.360, *P*_2-6_ = 0.117, *P*_3-4_ = 0.402, *P*_3-5_ = 0.576, and *P*_3-6_ = 0.802; *P*_4-5_ = 0.609, *P*_4-6_ = 0.276, and *P*_5-6_ = 0.564). There were no statistically significant differences between serum malondialdehyde concentrations of the control groups (groups 2 and 3) and the ALA, CoQ_10_, and ALA + CoQ_10_ groups but malondialdehyde levels of the ALA + CoQ_10_ group tended to be lower than those of the ALA and CoQ_10_ groups (*P*_2-4_ = 0.446, *P*_2-5_ = 0.420, *P*_2-6_ = 0.067, *P*_3-4_ = 0.372, *P*_3-5_ = 0.349, and *P*_3-6_ = 0.050; *P*_4-5_ = 0.965, *P*_4-6_ = 0.286, and *P*_5-6_ = 0.306). When compared with the control group, the ALA + CoQ10 group had lower serum malondialdehyde concentrations (*P* = 0.05).

## 4. Discussion

Ischemia refers to the decrease in blood supply of an organ which results in the breakdown of ATP and lipid peroxides so that the generation of lactic acid and hypoxanthine is enhanced. As blood supply normalizes during reperfusion, xanthine oxidase converts hypoxanthine to uric and superoxide radicals. These radicals consist of hydrogen peroxide, hydroxyl radicals, and superoxide anions. Superoxide radicals cause lipid peroxidation, which impairs the permeability of cell membranes, disrupt cellular integrity, and, thus, lead to cell damage. The production of nitric oxide and peroxynitrite is also accelerated in case of reperfusion followed by ischemia [20–23]. In this study, the apoptosis scores of the left ovaries were significantly lower than those of the right ovaries in the saline, olive oil, ALA, CoQ_10_, and ALA + CoQ_10_ groups. This finding indicates that a model of ischemia-reperfusion has been established successfully in all of the control and study groups.

ALA has been addressed as an efficient glutathione substitute which can increase cellular glutathione content and improve the antioxidant status of the myocardium [24]. It has been also reported that ALA protects against hepatic ischemia-reperfusion injury in rats [25]. The administration of ALA before the torsion of spermatic cord exerted significant protective effects against ischemia-reperfusion injury [26]. These protective effects may be attributed to the reduction of lipid peroxidation and the reinforcement of antioxidant defense mechanisms including glutathione and glutathione peroxidase [24–26].

A number of studies have focused on the utilization of CoQ_10_ for the prevention and treatment of ischemia-reperfusion injury in many organs. Kalayci et al. made up a model of brain ischemia-reperfusion injury and found that malondialdehyde levels were significantly reduced in rats that received a single dose of 10 mg/kg CoQ_10_ intraperitoneally [14]. Erol et al. concluded that CoQ_10_ treatment was able to decrease malondialdehyde levels in an experimental model of testicular ischemia-reperfusion injury [13]. Similarly, Ozler et al. reported that an intraperitoneal injection of CoQ_10_ could decrease oxidative stress markers significantly in a rat model of ovarian ischemia-reperfusion injury [19].

As for the present study, the administration of ALA or CoQ_10_ via oral route resulted in a decrease in apoptosis scores and an increase in NOS content of the ovaries that underwent torsion and subsequent detorsion. The NOS make up a group of enzymes that catalyze the production of nitric oxide from L-arginine. Nitric oxide is an important cellular signaling molecule that modulates the protection against oxidative damage. The increase in NOS content of the ovaries treated with ALA + CoQ_10_ indicates the antioxidant activity of the aforementioned molecules [27, 28].

In addition, ALA treatment caused an increase in serum glutathione peroxidase levels and a decrease in serum malondialdehyde concentrations. However, these alterations were not statistically significant. Such discrepancy may be the result of relatively smaller cohort size, oral route of administration for antioxidant molecules, relatively shorter follow-up period after surgery, and relatively insufficient dose of ALA administration. Oral route was preferred for the administration of antioxidant molecules because the availability and utility of these agents in clinical settings were a concern of this study. The other power-limiting factors were the inability to measure the tissue concentrations of oxidant and antioxidant molecules and the failure in specifying ovarian contents of endothelial and inducible NOS separately. These failures might be attributed to the relatively small size of prepubertal ovarian tissues.

The findings of the present study indicate a cumulative increase in NOS and a cumulative decrease in malondialdehyde levels for the combination of ALA and CoQ_10_. Yet, these alterations have been regarded as statistically insignificant. These findings imply that the combination of ALA and CoQ_10_ could improve ATP synthesis and reverse some of the cell damage caused by the reduction of energy availability. Moreover, this combination would extinguish the hazardous effects of oxidative stress which are primarily induced by the impairment of respiratory chain. An underlying factor for this synergetic effect might be that the combination treatment could diminish one or more of the final common pathways of mitochondrial dysfunction so that mitochondrial ATP production is improved [29, 30]. Another explanation may be the fact that the combination of two antioxidant agents would be more effective than an individual antioxidant agent because different antioxidants complement each other. Thus, there would be an increase in the total antioxidant capacity that is required to respond to the outburst of free radicals and reactive oxygen species [31].

Although the left ovaries have undergone torsion and subsequent detorsion in this study, the NOS content of the right ovary in the ALA + CoQ_10_ group has been found to be significantly higher. This finding can be explained by the reduction in blood supply of the right ovary. This reduction is caused by the vasoconstriction which is induced by the increase in the sympathetic activity after the torsion/detorsion procedure. On the other hand, no significant differences could be detected among the study groups in aspect of apoptosis. This failure could be due to the relatively small cohort size and the differences in the pathological examination techniques [32].

## 5. Conclusion

The findings of this study imply that the combination of ALA and CoQ_10_ has beneficial effects on oxidative stress induced by ischemia-reperfusion injury in an experimental model of ovarian torsion. These beneficial effects appear to be more pronounced within the ovarian tissue but less prominent in peripheral circulation. This finding may be due to the relatively shorter follow-up period after the administration of ALA or CoQ_10_.

Further research is warranted to clarify the effects of ALA and CoQ_10_ on the prepubertal ovarian tissues which have been exposed to ischemia-reperfusion injury.

## Figures and Tables

**Table 1:** Pathological scoring of all groups.

		Apoptosis score Mean ± std. deviation	*P*	Nitric oxide synthase Mean ± std. deviation	*P*	Histopathological evaluation Mean ± std. deviation	*P*
Group 1 (sham) (*n* = 7)	Left ovary	1.0 ± 0.0	0.259	0.2 ± 0.1	0.530	0.0 ± 0.0	1.00
	Right ovary	0.7 ± 0.2		0.3 ± 0.1		0.0 ± 0.0	

Group 2 Control (saline) (*n* = 7)	Left ovary	3.8 ± 0.9	0.003^*∗*^	1.3 ± 0.6	0.644	2.7 ± 0.48	0.001^*∗*^
	Right ovary	3.6 ± 0.7		1.7 ± 0.8		0.71 ± 0.48	

Group 3 Control (olive oil) (*n* = 7)	Left ovary	3.7 ± 0.5	0.002^*∗*^	1.7 ± 0.5	0.367	2.8 ± 0.37	0.001^*∗*^
	Right ovary	3.6 ± 0.7		2.0 ± 0.6		0.85 ± 0.69	

Group 4 (ALA) (*n* = 7)	Left ovary	2.7 ± 1.1	0.001^*∗*^	2.3 ± 1.1	0.709	1.7 ± 0.48	0.005^*∗*^
	Right ovary	0.6 ± 0.1		2.4 ± 1.3		0.57 ± 0.53	

Group 5 (CoQ_10_) (*n* = 7)	Left ovary	2.6 ± 1.4	0.002^*∗*^	2.4 ± 1.2	0.947	1.7 ± 0.75	0.015^*∗*^
	Right ovary	1.1 ± 0.3		2.1 ± 0.9		0.7 ± 0.48	

Group 6 (ALA + CoQ_10_) (*n* = 7)	Left ovary	2.5 ± 1.3	0.008^*∗*^	2.6 ± 1.4	0.578	1.7 ± 0.48	0.014^*∗*^
	Right ovary	0.9 ± 0.1		2.6 ± 1.5		0.57 ± 0.78	

^*∗*^ *P* < 0.05 was accepted to be statistically significant.
